# Diagnosis of Brugian Filariasis by Loop-Mediated Isothermal Amplification

**DOI:** 10.1371/journal.pntd.0001948

**Published:** 2012-12-13

**Authors:** Catherine B. Poole, Nathan A. Tanner, Yinhua Zhang, Thomas C. Evans, Clotilde K. S. Carlow

**Affiliations:** New England Biolabs, Ipswich, Massachusetts, United States of America; Queensland Institute for Medical Research, Australia

## Abstract

In this study we developed and evaluated a *Brugia Hha* I repeat loop-mediated isothermal amplification (LAMP) assay for the rapid detection of *Brugia* genomic DNA. Amplification was detected using turbidity or fluorescence as readouts. Reactions generated a turbidity threshold value or a clear visual positive within 30 minutes using purified genomic DNA equivalent to one microfilaria. Similar results were obtained using DNA isolated from blood samples containing *B. malayi* microfilariae. Amplification was specific to *B. malayi* and *B. timori*, as no turbidity was observed using DNA from the related filarial parasites *Wuchereria bancrofti*, *Onchocerca volvulus* or *Dirofilaria immitis*, or from human or mosquito. Furthermore, the assay was most robust using a new strand-displacing DNA polymerase termed *Bst 2.0* compared to wild-type *Bst* DNA polymerase, large fragment. The results indicate that the *Brugia Hha* I repeat LAMP assay is rapid, sensitive and *Brugia*-specific with the potential to be developed further as a field tool for diagnosis and mapping of brugian filariasis.

## Author Summary

Brugian filariasis is a debilitating neglected tropical disease caused by infection with the filarial parasites *Brugia malayi* or *Brugia timori*. Adult worms live in the lymphatic system and produce large numbers of microfilariae that predominantly circulate in the blood at night. Bloodsucking mosquitoes spread the disease by ingesting microfilariae that develop into infective stage larvae in the insect. In rural areas, diagnosis still relies largely on microscopic examination of night blood and morphological assessment of stained microfilariae. Loop-mediated isothermal amplification (LAMP) is a technique that can amplify DNA with high specificity, sensitivity and rapidity under isothermal conditions. The operational simplicity, versatility and low-cost of the technique make it particularly appealing for use in diagnosis and geographical mapping of neglected tropical diseases. In the present study, we have developed and evaluated a *Brugia Hha* I repeat LAMP assay for the rapid detection of *B. malayi* and *B. timori* genomic DNA. The results indicate that the *Brugia Hha* I repeat LAMP diagnostic assay is sensitive and rapid, detecting a single microfilariae in blood within 30 minutes, and *Brugia-*specific. The test has the potential to be developed further as a field tool for use in the implementation and management of mass drug administration programs for brugian filariasis.

## Introduction

Lymphatic filariasis is one of the world's most debilitating infectious diseases. According to the World Health Organization (WHO) (http://www.who.int/mediacentre/factsheets/fs102/en/), over 120 million people are currently infected in more than 80 countries. Approximately 40 million individuals are disfigured and incapacitated by the disease, including 15 million who have lymphoedema (elephantiasis) and 25 million men who have urogenital swelling, principally scrotal hydrocele. WHO estimates the loss of 5.1 million disability-adjusted life years (DALYs) as a result of infection by one of three filarial species (*Brugia malayi*, *Brugia timori* and *Wuchereria bancrofti*) [1]. Male and female parasites form “nests” in the lymphatic system, and after mating females produce large numbers of microfilariae that predominantly circulate in the blood at night. Microfilariae are ingested by a mosquito during a blood meal and develop to infective stage larvae that are subsequently transmitted to a new host.

In recent years there has been significant progress in the control of these diseases by the Global Programme to Eliminate Lymphatic Filariasis (GPELF) in which whole populations are treated by repeated, yearly cycles of mass drug administration (MDA) with antifilarial drugs [2], [3]. Over 2.6 billion treatments have been administered in 48 countries in the first 8 years [3], and this campaign continues to grow as new regions are included. Mapping of infected human and vector populations is required to identify areas in need of MDA. Following implementation, monitoring is necessary to determine the endpoint of treatment, with continued surveillance being required to identify areas of ongoing transmission or recrudescence. These activities and overall management of MDA programs are performed most efficiently with accurate diagnostic tools suitable for field use.

Point-of-care diagnosis of lymphatic filariasis is largely based on microscopic examination of night blood, and morphological assessment of stained microfilariae. A more accurate, rapid-format, immunochromatography card test (ICT) which detects circulating antigen is available for bancroftian filariasis [4], [5] but not for other filarial infections. Detection of microfilariae in conjunction with antibody testing, mainly in clinical settings, is being used as an interim measure for brugian filariasis [5]–[7]. However, the antibody tests indicate exposure rather than active infection [8], [9] and do not distinguish between bancroftian and brugian filariasis [10], thereby limiting their use for surveillance in areas where these infections are co-endemic.

Molecular-based diagnostic tools are considered more accurate since they detect active infection and have been used in laboratories for reliable differential identification of filarial parasites. Several polymerase chain reaction (PCR) based methods have been used to amplify DNA in blood from *B. malayi* and *B. timori* [11]–[16] and *W. bancrofti* [17]–[22]. Molecular monitoring of insect vectors by PCR is also the preferred method for xenodiagnosis and has been used extensively for *W.bancrofti* [20]–[29] and to a lesser extent for *B. malayi* [27], [30]–[32]. PCR however, requires highly skilled personnel and expensive equipment.

An alternative to PCR, is a technique termed loop-mediated isothermal amplification (LAMP) which amplifies DNA with high specificity, sensitivity and rapidity under isothermal conditions using a polymerase with strand displacement activity. The enzyme generates a mixture of stem-loops containing alternately inverted repeats of the target sequence and cauliflower-like structures resulting in exponential amplification of the target sequence (>10 µg, >50× PCR yield) [33]–[35]. The LAMP reaction uses two sets of primers, outer primers (F3 and B3) and inner primers (FIP and BIP) that hybridize to six sites on the target DNA. Specially designed FIP and BIP primers each consisting of two distinct sequences correspond to sense and antisense sites on the target DNA. The addition of a third set of primers, known as loop primers, has been shown to accelerate the reaction [34]. Using three primer sets recognizing eight sites in the target DNA lends LAMP the specificity to discriminate between genomic DNA at both genus- and species-specific levels [36], [37]. In recent years this technology has been explored for the diagnosis of certain parasitic [38]–[45], bacterial [46], fungal [47] and viral [48], [49] infections. Because of its simplicity, rapidity, and versatility in readout options, LAMP offers a distinct advantage over other molecular diagnostic methods for use in the field. LAMP test kits are now commercially available or in development for the detection of *Mycobacterium tuberculosis* complex [50], and human African trypanosomiasis [52] for use in resource-limited settings.

In the present study we report on the development of a simple LAMP test that amplifies the *Brugia*-specific *Hha* I repeat for the rapid detection of *B. malayi or B. timori* DNA. We evaluated the efficacy of several thermophilic DNA polymerases using real-time LAMP, and also compared read-out options. Our results demonstrate that the *Hha I* LAMP test is sensitive and specific with the potential to be developed further as a field tool for diagnosis and mapping of brugian filariasis.

## Materials and Methods

### Reagents

DNA samples were generously donated by the following: *B. malayi* and *B. timori*, L.A. McReynolds (New England Biolabs); *Onchocerca volvulus* and *Homo sapiens*, F. Perler (New England Biolabs); *Dirofilaria immitis*, C. Maina (New England Biolabs); and *Aedes albopictus*, Z. Li (New England Biolabs). Whole genome amplified *Wuchereria bancrofti* DNA, heparinized *B. malayi* infected feline blood and uninfected dog blood were obtained from the NIH/NIAID Filariasis Research Reagent Resource Center (http://www.filariasiscenter.org). The purity and quantity of DNA in samples was determined using a Nano Drop Spectrophotometer, ND-1000 (Nano Drop Technologies).

### Preparation and Processing of *B. malayi* Infected Blood Samples

A two-fold dilution series of *B. malayi* microfilaraemic feline blood was diluted using uninfected dog blood. Forty µl aliquots of each dilution were used for quantifying microfilarial titers and the same volume used for DNA extraction. Microfilarial counts were determined using a membrane concentration technique [53], [54]. Briefly, 40 µl aliquots of each dilution were mixed with 160 µl PBS then filtered through a 5.0 µm pore polycarbonate membrane (Nucleopore, Whatman). Filters were placed, microfilariae side up, on a microscope slide and stained [55], [56]. Parasites were counted using an Axio Scope A1 (Zeiss) at 40× magnification. DNA was extracted from 40 µl of each dilution using a QIAamp DNA Mini Kit (Qiagen) after digesting the samples with proteinase K in the supplied AL Buffer for 2 hrs at 56°C. Purified DNA was eluted in a 200 µl volume; one µl of which was used in LAMP assays.

### Primer Design

**Figure 1:**
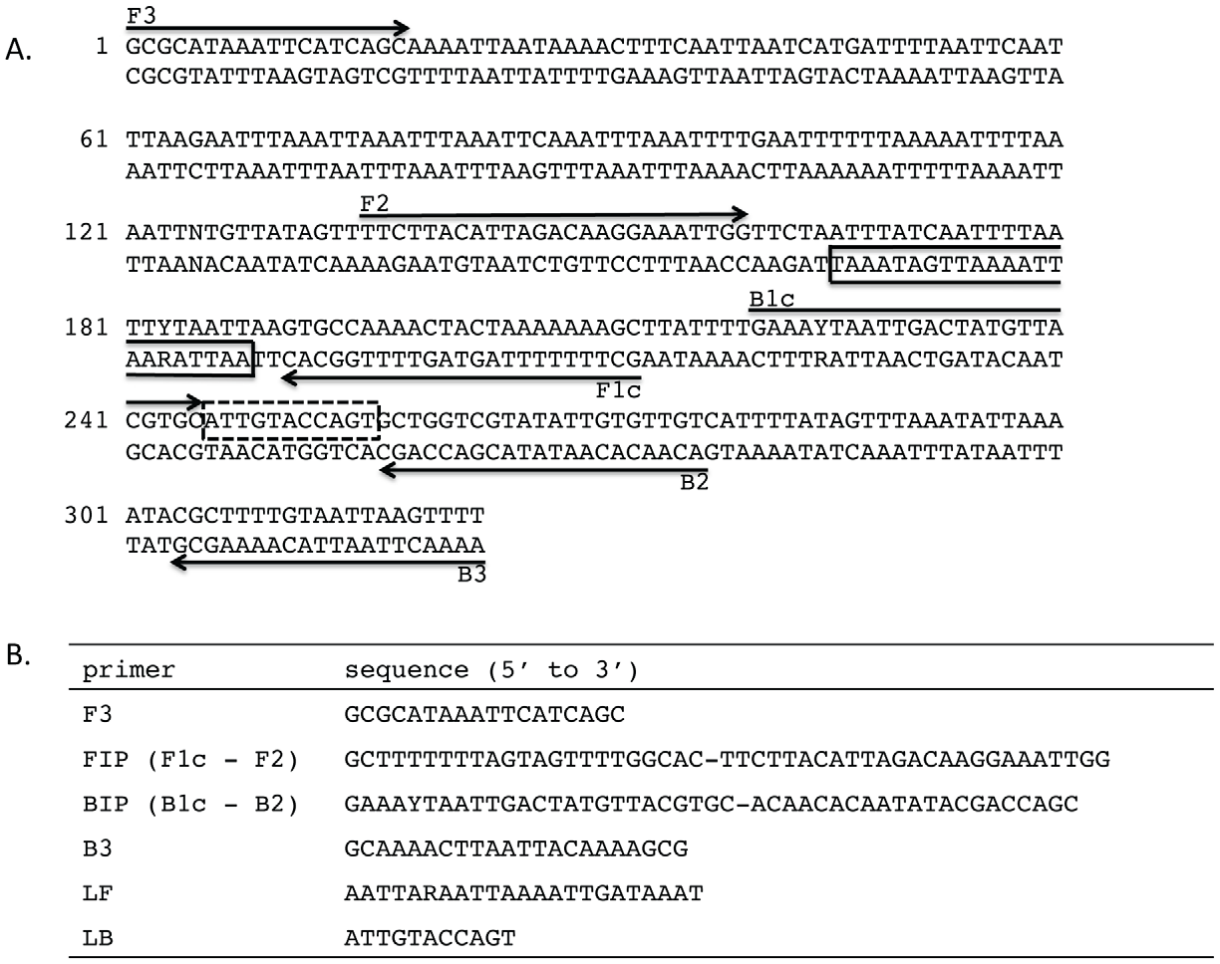
LAMP primer set targeting the *B. malayi Hha* I repeat family. (A) The location of the six LAMP primers within the sequence of the consensus *Hha* I repeat is shown. Arrows indicate the direction of extension. The solid and dash line boxes represent the binding regions of the loop forward (LF) and loop back (LB) primers respectively. (B) Sequence of the consensus *Hha* I repeat LAMP primers. The GC dinucleotide at the 5′ end of B3 is derived from the first two nucleotides of the repeat and is added to raise the GC content of the oligonucleotide. BIP and LF are degenerate oligonucleotides where Y = C or T and R = A or G.

To generate primers for LAMP, multiple *B. malayi Hha* I repeat sequences were aligned using ClustalW [57]. Accession number M12691 [58] was used to query the *B. malayi* whole-genome shotgun (WGS) database at GenBank using the blastn program (http://blast.ncbi.nlm.nih.gov). Full-length repeats were selected from accession numbers: M12691, AAQA01025653, AAQA01026145, AAQA01018878, AAQA01011954, AAQA01021048, AAQA01005386, AAQA01005790, AAQA01007277, AAQA01004714, AAQA01005124, and used to generate a consensus sequence (Figure 1A and Figure S1). LAMP primers (Figure 1B) were designed manually using “A guide to LAMP primer design” available from the Eiken Chemical Co. (http://primerexplorer.jp/e/).

To facilitate the design of PCR primers for amplification of actin, sequences from *B. malayi* (NW_001892317.1, region: 12826–14482; NW_001893014.1, region: complement 253210–256438), *O. volvulus* (M84915, M84916), *W. bancrofti* (AF184961), *Aedes aegypti* (NW_001810656, region: complement (1446599–1449390) and *Homo sapiens* (NC_000001, region: complement 229569843–229566992) were downloaded from GenBank and aligned using ClustalW [57]. The region corresponding to exon 2 in the *W. bancrofti* and *O. volvulus* actin genes [59], [60], that also exhibited high identity among all members in the alignment, was used to design degenerate PCR primers. The forward and reverse primer sequences are (5′ GCTCAGTCBAAGAGAGGTAT 3′) and (5′ACAGCYTGGATDGCAACGTACA 3′), respectively, where B = C, G or T; Y = C or T, and D = A, G or T. PCR and LAMP primers were synthesized by Integrated DNA Technologies (Coralville, Iowa).

### LAMP Assays

LAMP reactions with *Bst* DNA polymerase, large fragment (LF, New England Biolabs) contained 1.6 µM each of FIP and BIP, 0.2 µM each of F3 and B3, 1.4 mM of each dNTP, 20 mM Tris-HCl (pH 8.8), 10 mM KCl, 10 mM (NH_4_)_2_SO_4_, 8 mM MgSO_4_, 0.1% Tween-20 and 8 U of enzyme mixed with 1 µl of various genomic DNAs in a total volume of 25 µl. For the evaluation of the *Hha* I LAMP primer set with either *Bst* 2.0 DNA polymerase or *Bst* 2.0 WarmStart DNA polymerase (New England Biolabs), reactions were set up and performed as described above, except 50 mM KCl was used. Loop Forward (LF) and Loop Back (LB) primers were added to some reactions (0.4 µM) to assess their ability to decrease the threshold time under various conditions. Reactions were incubated at 63°C for 60–90 minutes in a Loopamp Realtime Turbidimeter (LA-320c, Eiken Chemical Co.). The instrument measures the change in turbidity at 650 nm caused by the precipitation of magnesium pyrophosphate with time. Turbidity data were analyzed using the LA-320c software package that reports when the change in turbidity over time (dT/dt) reaches a value of 0.1, which we then assigned to be the threshold time (Tt). When amplification was evaluated using the calcein-based Fluorescent Detection Reagent ([61] and the Eiken chemical Co.) rather than turbidity, reactions were heat killed for 20 min at 80°C then visualized within 60 minutes with UV light at 365 nm as recommended by the manufacturer.

### PCR Assay

As a positive control for the presence of intact DNA, a 244 bp actin fragment was PCR amplified from various genomic DNAs using 1.25 U of *Taq* DNA polymerase in 1× standard buffer (New England Biolabs) containing 3.5 mM MgCl_2_, 0.2 mM each dNTP, and 0.2 µM each of the forward and reverse actin primers in a 50 µl reaction. One ng of genomic DNA was used as template, except for *B. timori* (5 ng) and human (10 ng). Reactions containing human DNA, contained 4 mM MgCl_2_. All reactions was denatured once at 95°C for 30 sec then cycled 30 times at 95°C for 30 sec, 55°C for 30 sec and 68°C for 30 sec, except 35 cycles were used for *B. timori* and non-template controls. After cycling, reactions were incubated for 5 min at 68°C then the reaction products were analyzed by electrophoresis using 1.2% agarose gels equilibrated with TBE buffer.

## Results

### The *B. malayi Hha I* LAMP Assay Is Sensitive

**Figure 2:**
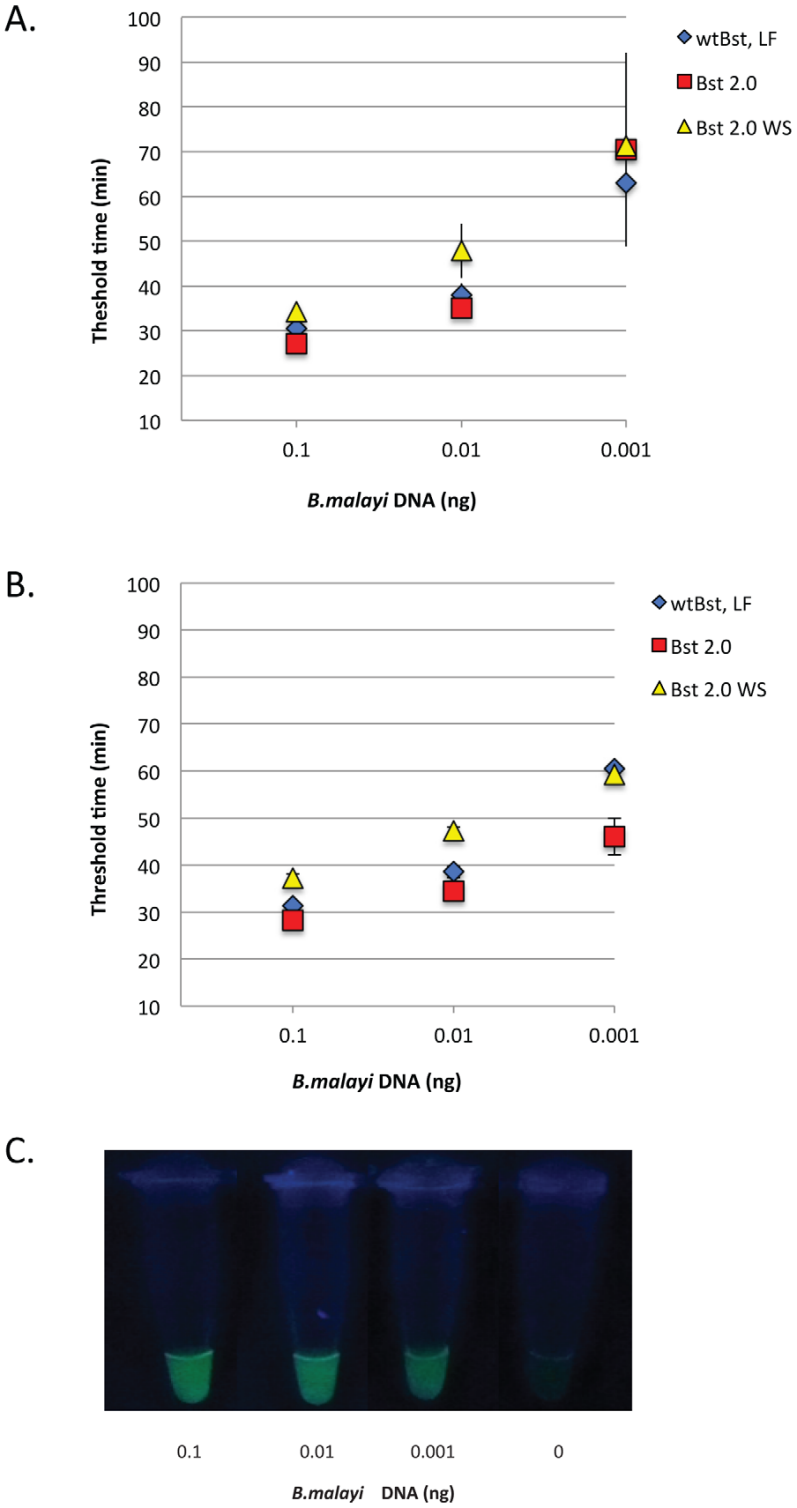
Sensitivity of *Hha* I LAMP assay. Ten-fold serial dilutions of *B. malayi* genomic DNA amplified with the *Hha* I primer set alone (A) or in the presence of loop primers (B) with *Bst* DNA polymerase, large fragment (wt *Bst* LF), *Bst* 2.0 DNA polymerase (*Bst* 2.0) and *Bst* 2.0 WarmStart DNA polymerase (*Bst* 2.0 WS). Data points represent the average of three samples and the error bars represent the standard deviation at each point. For each enzyme, the average threshold time, defined as the time at which the change in turbidity over time (dT/dt) reaches a value of 0.1, is plotted against the amount of starting material. (C) UV detection (365 nm) of products generated within 60 minutes using *Bst* 2.0 in the presence of loop primers and Fluorescent Detection Reagent. The amount of starting material in ng is shown below the photograph. Positive samples fluoresce green while negative samples remain dark.

To maximize assay sensitivity, the *B. malayi Hha* I repeat was selected as a target for amplification because of its abundance in the genome [58]. A *B. malayi Hha* I consensus sequence derived by aligning 34 repeats was used to design a primer set for LAMP (Figure 1 and Figure S1). Primers were designed manually as the AT richness (79%) of the consensus sequence precluded use of the Primer Explorer software (http://primerexplorer.jp/e/) for LAMP primer design. The sensitivity of the *Hha* I primer set was evaluated by real time turbidity using three thermophilic DNA polymerases, *Bst* DNA polymerase, LF, *Bst* 2.0 DNA polymerase or *Bst* 2.0 WarmStart DNA polymerase. Ten-fold serial dilutions of genomic *B. malayi* DNA ranging from 0.1–0.001 ng were amplified using both the *Hha* I primer set alone (Figure 2A) and in the presence of loop primers (Figure 2B). At the highest concentration of template DNA (0.1 ng), reactions reached a turbidity threshold of 0.1 in approximately 30 minutes regardless of the polymerase employed (Figure 2A). As the concentration of template DNA decreased, there was a corresponding increase in the amount of time required to reach the threshold value of 0.1. Reactions using *Bst* 2.0 DNA polymerase improved the most with reliable detection of 0.001 ng parasite DNA, corresponding to 1/100^th^ of a microfilariae, within 45 minutes compared to ∼70 minutes without loop primers (Figure 2A and 2B). In the absence of template or primers, no turbidity was observed. Likewise at concentrations of template ≤0.0001 ng, only one or none of the triplicate samples amplified (data not shown). Reaction times were slightly slower using *Bst* 2.0 WarmStart DNA polymerase regardless of the presence of loop primers (Figure 2A and 2B). Similar sensitivity was obtained within the same time frame when the calcein-based Fluorescent Detection Reagent rather than turbidity was used as the output. Positive reactions turned green while no color change was apparent in the absence of amplification or when no target DNA (or ≤0.0001 ng) was present (Figure 2C).

**Figure 3:**
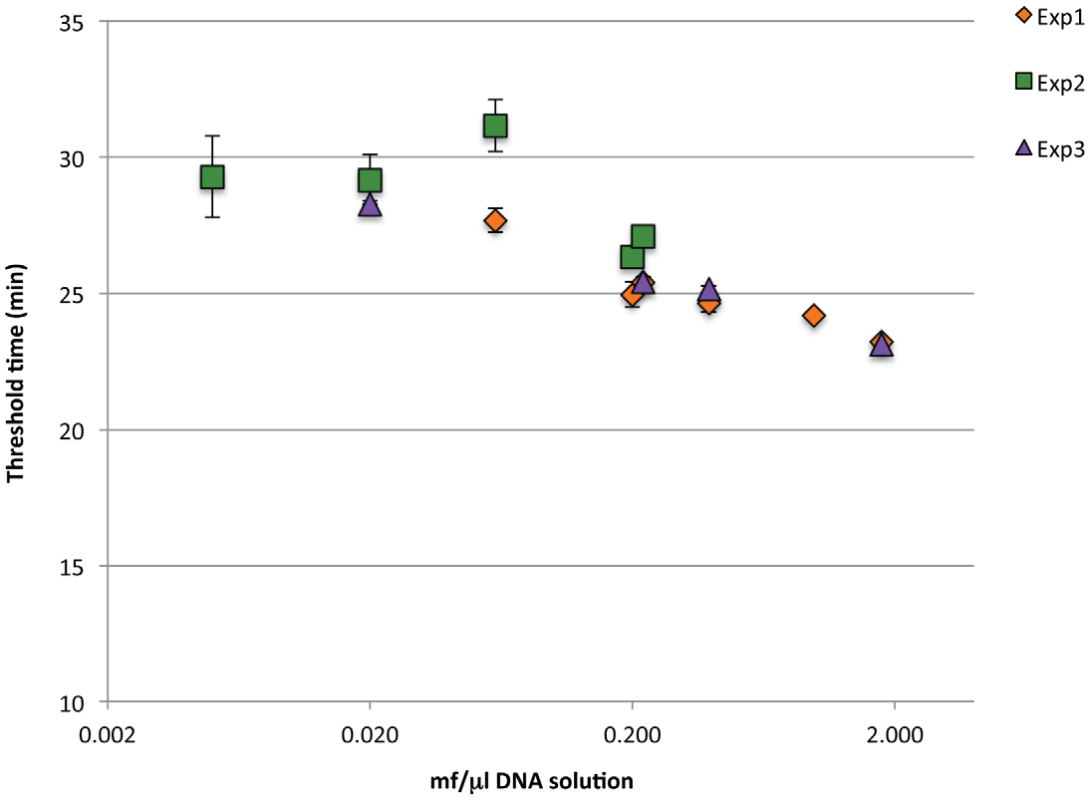
*Hha* I LAMP assay for the detection of *B. malayi* infected blood samples. A set of serial dilutions (two-fold) of microfilariae in blood was prepared and DNA was isolated from each dilution. Three experiments were performed using a different but overlapping range of DNA dilutions. One µl of DNA from each dilution was used in LAMP reactions with *Bst* 2.0 DNA polymerase. Samples from each experimental set-up were performed in triplicate (experiments 1 and 2) or duplicate (experiment 3). Average threshold times and standard deviations were plotted against the approximate number of mf/µl DNA solution.

To mimic a clinical situation, the assay was performed using *Bst* 2.0 DNA polymerase on DNA extracted from a two-fold dilution series equivalent to 25–9000 mf/ml blood. Three experiments were performed using a different but overlapping range of DNA dilutions equivalent to 1/200^th^-2 microfilariae per LAMP reaction. Good concordance was observed between samples containing equivalent amounts of template DNA. A turbidity threshold of 0.1 was reached in 25–30 minutes with slightly more time required (<5 minutes) as the concentration of template DNA decreased (Figure 3). No turbidity was detected when uninfected blood was processed in the same manner (data not shown).

### Evaluation of Assay Specificity

**Figure 4:**
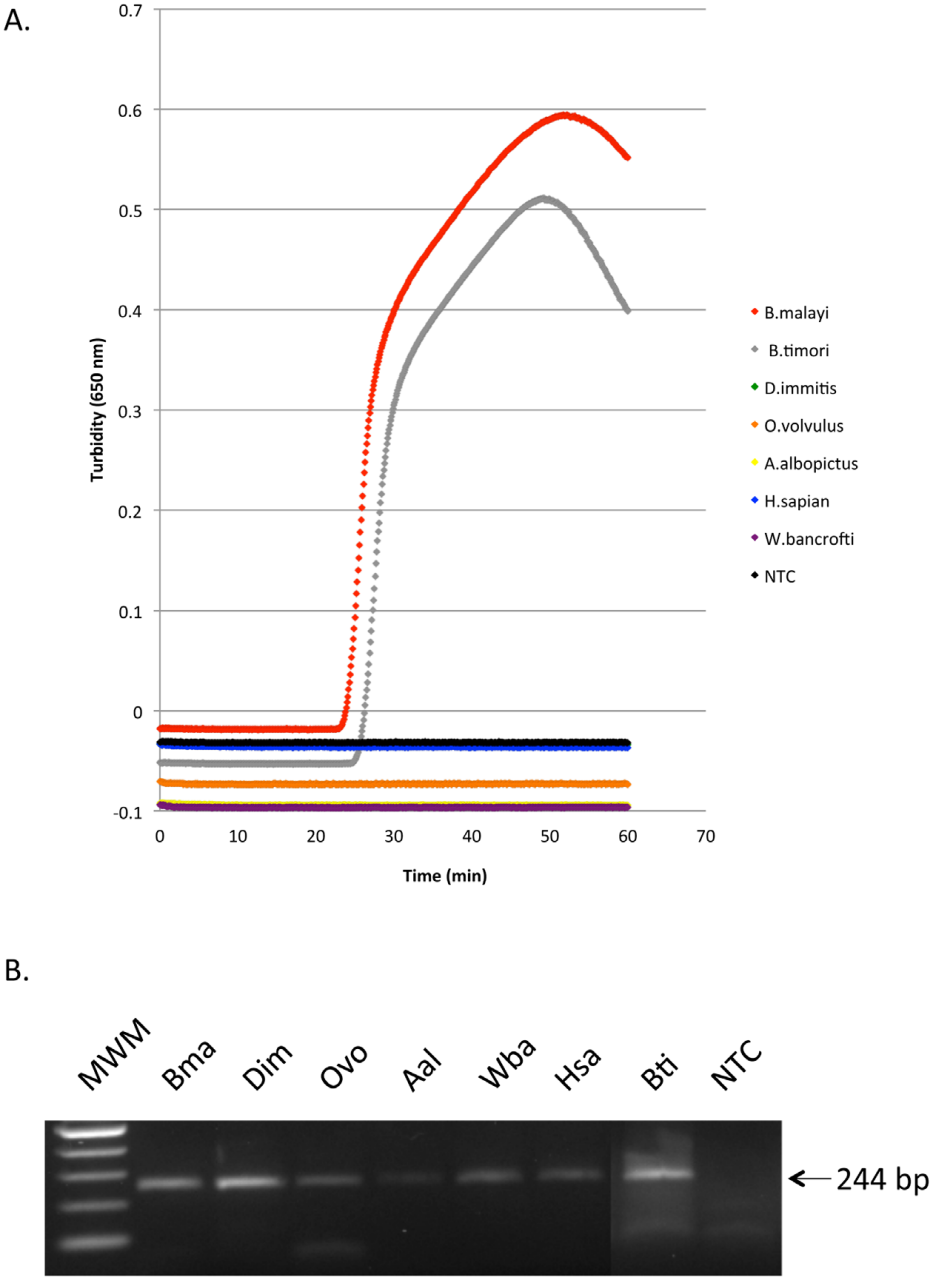
Species-specificity of *Hha* I LAMP assay. (A) Each curve represents the calculated average of triplicate turbidity curves generated with various genomic DNAs (0. 1 ng) using *Bst* 2.0 DNA polymerase without loop primers. Turbidity was observed using *B. malayi* or *B. timori* DNA. (B) As a positive control, an actin gene fragment was PCR amplified from *B. malayi* (Bma), *D. immitis* (Dim), *O. volvulus* (Ovo), the mosquito *Aedes albopictus* (Aal), *W. bancrofti* (Wba), human (Hsa) and *B. timori* (Bti) DNAs using degenerate primers. Agarose gel showing amplification of a 244 bp fragment of the actin gene. The 100 bp DNA Ladder (New England Biolabs) was used as the molecular weight marker (MWM). Water was used in the non-template controls (NTC) in (A) and (B).

We evaluated the performance of LAMP for the differential detection of the *Hha* I repeat in genomic DNA samples isolated from the closely related parasites *B. timori*, *W. bancrofti*, *D. immitis*, and *O. volvulus*. DNA from human and mosquito (*Aedes albopictus*) samples and a non-template control were also included for comparison. As observed in previous experiments, turbidity reached a threshold value of 0.1 in approximately 30 minutes when 0.1 ng of *B. malayi* or *B. timori* DNA was added to the reaction, whereas no turbidity was observed when DNA from the other filarial parasites, human or mosquito was used (Figure 4A). The integrity of these various DNAs was confirmed in PCR experiments using primers designed to amplify an actin gene. A single amplification product of 244 bp, the expected fragment size was obtained (Figure 4B).

## Discussion

The *Brugia Hha* I repeat was selected as the biomarker for a LAMP-based diagnostic test for brugian filariasis. The repeats are non-protein coding, approximately 322 bp in length, and arranged in direct tandem arrays. They comprise between 1–12% of the *B. malayi* genome [58], [62] and are highly conserved, with the nucleotide identity of the repeats used in this study varying from 82–98%. In order to target the greatest number of repeats and maximize assay sensitivity, primers were designed based on the most highly conserved nucleotide blocks in a consensus sequence. The *Hha* I PCR amplification system has been shown to be extremely sensitive in detecting *Brugia* DNA [11], [63], exceeding the theoretical limit of detection of one microfilaria per ml using conventional microscopy and concentration techniques [64].

There are several important advantages offered by LAMP over PCR. Its operational simplicity and isothermal nature make it ideally suited for use in the field. In PCR, thermal cycling is required to denature the template, anneal primers and extend the amplicon. LAMP employs *Bst* DNA polymerase, LF which provides both strand displacement and target amplification at a single temperature in a simple heat block or water bath at 60–65°C [33]. High levels of sensitivity and specificity can be achieved in LAMP because the amplification reaction involves four specific oligonucleotide primers that anneal to six distinct regions within the target sequence [33]. Also, the addition of loop primers may further improve performance [34]. Levels of sensitivity comparable to the *Hha* I PCR amplification system were obtained in the *Hha* I LAMP test using either DNA isolated from worms or from blood containing microfilariae. It is estimated that a single microfilaria contains approximately 100 pg of DNA [11], [14], [21] and using the LAMP *Hha* I test it is possible to easily detect as little as 1 pg of total genomic DNA purified from *B. malayi* worms which is equivalent to 1/100^th^ of a microfilaria. In mock experiments using DNA prepared from a dilution series of microfilaremic blood, we detected the equivalent of 1/200th of a microfilaria in approximately 30 minutes. This was the most dilute DNA sample tested and is equivalent to one mf in 40 µl of whole blood. It is possible that free DNA contributed to the output signal when using blood since it has been suggested that 130 fg of repeat can be released by a single dead microfilaria [63]. In a *Hha* I based PCR-ELISA, free DNA of nocturnally periodic *B. malayi* was detected in 200 µl of day blood, achieving a sensitivity comparable with filtration of 1 ml of night blood [63].

In the present study, LAMP reaction times were fastest at lowest DNA concentrations of template using a new isothermal strand-displacing polymerase *Bst* 2.0 DNA polymerase in the presence of loop primers. *Bst* 2.0 DNA polymerase is an *in silico* designed homologue of *Bst* DNA polymerase, LF engineered for improved amplification speed, yield, salt tolerance and thermostability [65]. Its warmstart version (*Bst* 2.0 WarmStart DNA polymerase) possesses a reversibly-bound aptamer which inhibits polymerase activity at temperatures below 45°C. This circumvents a common problem that can occur in nucleic acid amplification namely the undesired activity from DNA polymerases during room temperature reaction set-up [65]–[67]. In our experiments, reaction times were slightly slower using *Bst* 2.0 WarmStart DNA polymerase due to the presence of the aptamer. In assays designed to mimic field conditions, *Bst* 2.0 WarmStart DNA polymerase enables amplification of the *Hha* I repeat without generating a signal in the non-template controls when incubated at 35°C for intervals up to 2 hrs before initiating amplification, in contrast to *Bst* DNA polymerase, LF and *Bst* 2.0 DNA polymerase (data not shown). The ability to allow LAMP reactions to be assembled and stored at room temperature for hours with no change in the final readout can offer a distinct advantage in resource-limited settings.

In addition to sensitivity, the *Hha* I LAMP test offers the high level of specificity required for diagnosis and mapping. The *Hha* I LAMP primer amplified *B. malayi* and *B. timori* DNA but not DNA isolated from the closely related filarial parasites *W. bancrofti*, *D. immitis*, or *O. volvulus*, or from human or mosquito. Previous studies have shown that the *Hha* I repeat family in *B. timori* is highly homologous to the *B. malayi Hha* I repeat family [15], [68]. Therefore the test may be useful for diagnosing infection in patient samples and monitoring transmission of *B. malayi* or *B. timori* in mosquito vectors. Recently species-specific primers have also been used in LAMP to detect DNA from *D. immitis* [69], [70] and *W. bancrofti* [43] in blood and mosquito samples.

Rapidity and versatility in readout options also make LAMP a particularly appealing technology. Positive results can be visualized by turbidity caused by precipitation of magnesium pyrophosphate, a by-product of the reaction that can be seen with the naked eye [71]–[73] within 15–60 minutes. The reaction product can also be detected under UV light with the addition of fluorescent dyes [72], [74]–[79] or colorimetrically using hydroxy naphthol [80]. In the present study, real-time turbidity was used for assay design and optimization. The time at which the reaction reaches a threshold of 0.1 turbidity was used to precisely evaluate various parameters. Similar results were obtained when calcein [61] was added to reactions. In addition, recent estimates suggest that diagnostic LAMP tests are significantly cheaper than PCR. The estimated cost of a *W. bancrofti* LAMP test is $0.82 compared with more than $2.20 for PCR [43].

The operational simplicity of the LAMP technique makes it particularly appealing for neglected tropical diseases, as evidenced by the rate of adoption of this diagnostic DNA technology by laboratories in developing countries. Since many of these diseases are co-endemic, it is desirable to leverage resources and integrate diagnostic platforms wherever possible. Multiplexing of the LAMP reaction has been demonstrated for the detection of *Babesia* parasites in cattle [37] malaria and heartworm in mosquitoes [69] and human arboviruses [81]. More recently a real-time, multiplex LAMP technique was described that enables detection of up to 4 distinct LAMP targets in a single reaction [65].

In summary, we describe a promising *Hha* I-based LAMP diagnostic assay for *brugian filariasis* using *Bst* 2.0 DNA polymerase and loop primers that generates a robust read-out within 60 minutes. The assay warrants further testing with endemic samples as the next stage in development towards its use as a field tool for implementation and management of MDA programs.

## Supporting Information

Figure S1 **Alignment of** ***B. malayi Hha*** **I repeat sequences.** Full-length *Hha* I repeat DNA sequences were obtained from the following GenBank accession numbers: *Hha* I_1, M12691; *Hha* I_2a, AAQA01025653; *Hha* I_3a, AAQA01026145; *Hha* I_4a and 4b, AAQA01018878; *Hha* I_5a–5c, AAQA01011954; *Hha* I_6a and 6b, AAQA01021048; *Hha* I_7a–7c, AAQA01005386; *Hha* I_8a–8d, AAQA01005790; *Hha* I_9a–9d, AAQA01007277; *Hha* I_10a–10f, AAQA01004714; *Hha* I_11a–11e, AAQA01005124. RC denotes that the reverse complement of the sequence was used. The consensus sequence used for LAMP primer design is shown above the alignment. **GenBank accession numbers in this manuscript**: M12691, AAQA01025653, AAQA01026145, AAQA01018878, AAQA01011954, AAQA01021048, AAQA01005386, AAQA01005790, AAQA01007277, AAQA01004714, AAQA01005124, NW_001892317.1, NW_001893014.1, M84915, M84916, AF184961, NW_001810656, NC_000001.
(TIF)
