# Supplementary figures and images for: Diagnosis of Brugian Filariasis by Loop-Mediated Isothermal Amplification
Source: PLoS Negl Trop Dis. 2012 Dec 13;6(12):e1948. doi: 10.1371/journal.pntd.0001948 (PMC3521703; doi:10.1371/journal.pntd.0001948)

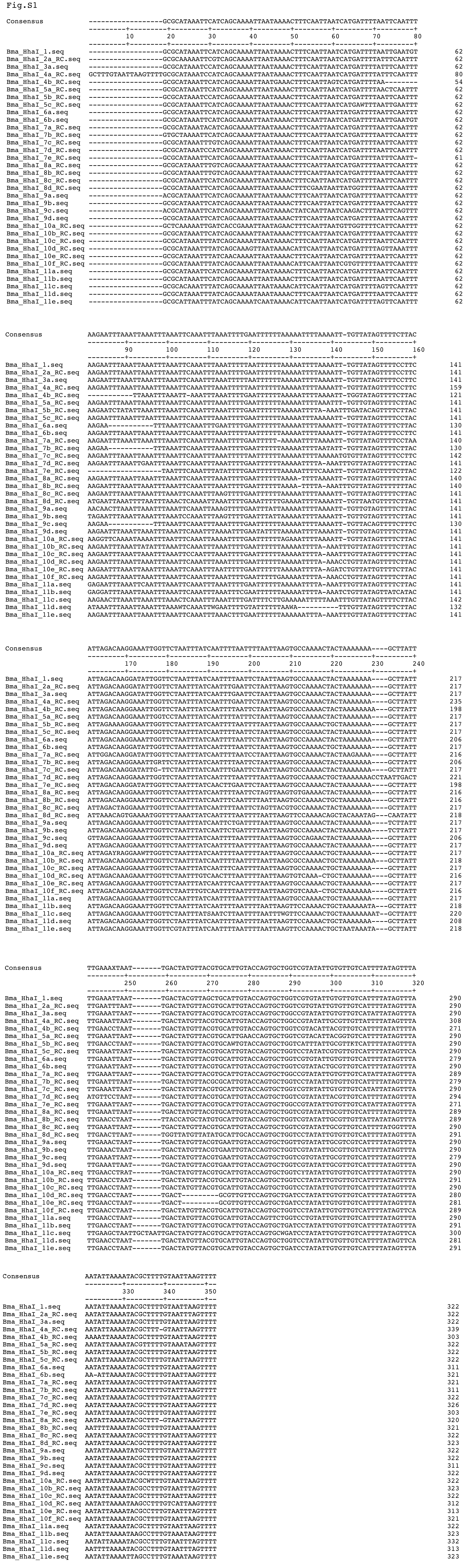

Supplement: Figure S1 — Alignment of B. malayi Hha I repeat sequences. Full-length Hha I repeat DNA sequences were obtained from the following GenBank accession numbers: Hha I_1, M12691; Hha I_2a, AAQA01025653; Hha I_3a, AAQA01026145; Hha I_4a and 4b, AAQA01018878; Hha I_5a–5c, AAQA01011954; Hha I_6a and 6b, AAQA01021048; Hha I_7a–7c, AAQA01005386; Hha I_8a–8d, AAQA01005790; Hha I_9a–9d, AAQA01007277; Hha I_10a–10f, AAQA01004714; Hha I_11a–11e, AAQA01005124. RC denotes that the reverse complement of the sequence was used. The consensus sequence used for LAMP primer design is shown above the alignment. GenBank accession numbers in this manuscript: M12691, AAQA01025653, AAQA01026145, AAQA01018878, AAQA01011954, AAQA01021048, AAQA01005386, AAQA01005790, AAQA01007277, AAQA01004714, AAQA01005124, NW_001892317.1, NW_001893014.1, M84915, M84916, AF184961, NW_001810656, NC_000001. (TIF) [file pntd.0001948.s001.tif]
